# Targeting Wnt-driven metabolic adaptations in cancer: integrating glycolysis, glutaminolysis, IDO1-mediated immune evasion, and therapeutic delivery strategies

**DOI:** 10.3389/fcell.2025.1622218

**Published:** 2025-08-22

**Authors:** Eric Flores-Hernández, Grace Binder, Kuo-Ching Mei, Nydia Tejeda-Muñoz

**Affiliations:** ^1^ Departamento de Biología Celular, Facultad de Ciencias, Universidad Nacional Autónoma de México (UNAM), Mexico City, Mexico; ^2^ Department of Biological Chemistry, David Geffen School of Medicine, University of California, Los Angeles, Los Angeles, CA, United States; ^3^ Department of Pharmaceutical Sciences, School of Pharmacy and Pharmaceutical Sciences, State University of New York at Binghamton, Binghamton, NY, United States; ^4^ Department of Molecular Pharmaceutics, College of Pharmacy, University of Utah, Salt Lake City, UT, United States; ^5^ Department of Oncology Science, University of Oklahoma Health Sciences Center, Oklahoma City, OK, United States; ^6^ OU Health Stephenson Cancer Center, University of Oklahoma Health Sciences Center, Oklahoma City, OK, United States

**Keywords:** Wnt signaling, macropinocytosis, metabolic reprogramming, colorectal cancer, membrane trafficking, targeted cancer therapies

## Abstract

The Wnt pathway is an evolutionarily conserved signaling cascade that regulates a wide range of fundamental cellular processes, including proliferation, differentiation, polarity, migration, metabolism, and survival. Due to its central regulatory roles, Wnt signaling is critically involved in the pathophysiology of numerous human diseases. Aberrant activation or insufficient inhibition of this pathway has been causally linked to cancer, degenerative disorders, metabolic syndromes, and developmental abnormalities. Wnt signaling drives cancer progression by reprogramming metabolism and promoting immune evasion. Wnt-driven tumors exhibit enhanced aerobic glycolysis (the Warburg effect), glutaminolysis, and macropinocytosis, which support rapid proliferation and help maintain redox homeostasis under nutrient-limited or nutrient-deprived conditions. These metabolic adaptations sustain tumor survival and contribute to immune suppression, as seen in the Wnt5a-indoleamine 2,3-dioxygenase 1 (IDO1) axis, which fosters regulatory T-cell expansion and an immunosuppressive microenvironment. The interplay among glycolysis, glutamine metabolism, and immune escape renders Wnt-driven cancers highly adaptable and resistant to conventional therapies. Targeting metabolic enzymes, such as pyruvate dehydrogenase kinase 1 (PDK1), lactate dehydrogenase A (LDHA), glutaminase (GLS), and monocarboxylate transporters (MCT-1), alongside immune checkpoint inhibitors or IDO1 blockade, presents a promising strategy for overcoming metabolic plasticity and immune evasion in Wnt-driven malignancies, thereby enhancing therapeutic efficacy and improving patient survival in otherwise refractory tumor types. Combining glycolysis and glutaminolysis inhibitors with T-cell activating therapies may disrupt tumor metabolic plasticity and restore anti-tumor immunity. Additionally, advanced drug delivery systems, including lipid nanoparticles (LNPs), polymeric nanocarriers, and exosome-based platforms, enhance the targeted accumulation of metabolic inhibitors and immunomodulatory agents while minimizing systemic toxicity. This review examines the metabolic and immune adaptations of Wnt-driven cancers, with a focus on glycolysis, glutaminolysis, and macropinocytosis. We highlight emerging therapeutic targets and nanomedicine-based delivery strategies to counteract metabolic adaptation and immune suppression. By integrating metabolic and immune-targeting with precision nano-delivery platforms, future treatment paradigms may improve outcomes for aggressive and therapy-resistant Wnt-driven cancers.

## Introduction

A significant shift occurs in the metabolic state of cells during cancer progression which involves the metabolism of carbohydrates, lipids, and amino acids (Kimmelman and White, 2017). This metabolic reprogramming enables the tumor to supply itself with the necessary nutrients and energy to drive proliferation, angiogenesis, and overall tumor progression (Martínez-Reyes and Chandel, 2021). One of the key metabolic adaptations in tumor cells is the Warburg effect, also known as aerobic glycolysis, which is characterized by an increased glycolytic index, elevated lactate production, and decreased oxidative phosphorylation, even in the presence of oxygen and fully functional mitochondria (Warburg, 1956). While glycolysis is less efficient in adenosine triphosphate (ATP) production than mitochondrial respiration, it facilitates biosynthetic processes essential for tumor progression including nicotinamide adenine dinucleotide phosphate (NADPH) production, lipid synthesis, and the generation of non-essential amino acids (Vaupel and Multhoff, 2021).

In colorectal cancer (CRC), Wnt signaling is a key driver of tumor progression, with its dysregulation commonly occurring due to Adenomatous Polyposis Coli (APC) mutations that stabilize β-catenin, leading to the constitutive activation of Wnt target genes (Fu et al., 2018; Liu and Yin, 2017). Beyond its role in proliferation and stemness, Wnt signaling has emerged as a central regulator of metabolic reprogramming in cancer cells (Table 1). Activating canonical (β-catenin-dependent) Wnt signaling enhances glycolysis by upregulation of key enzymes such as pyruvate dehydrogenase kinase 1 (PDK1), pyruvate kinase M2 (PKM2), lactate dehydrogenase A (LDH-A), monocarboxylate transporters (MCT-1), and glucose transporters (GLUT) (Roche et al., 2001). Inhibition of Wnt signaling, by targeting PDK1 with Peroxisome proliferator-activated receptor gamma coactivator 1-alpha (PGC1α) or Chibby (a β-catenin antagonist) has been shown to reduce glycolytic activity, leading to impaired tumor growth and altered metabolic dependencies (Pate et al., 2014; Zuo et al., 2021; Cai et al., 2018). However, in addition to glycolysis, Wnt-driven tumors exhibit metabolic plasticity, shifting towards glutaminolysis to sustain biosynthetic needs. Glutaminolysis supports Tricarboxylic Acid (TCA) cycle replenishment, redox balance, and epigenetic modifications that enhance tumor progression (Dang et al., 2010; Seltzer et al., 2010). This dual reliance on glycolysis and glutaminolysis makes Wnt-driven tumors metabolically adapt, allowing them to thrive even under fluctuating nutrient conditions (Park et al., 2023; Vallée et al., 2021).

**TABLE 1:** Metabolic effects of Wnt/β-catenin signaling pathway modulation.

Cause	Metabolic proteins involved	Effect	References
Blocking of Wnt/β-catenin signaling by dnLEF/TCFs or XAV939	PDK1	Reduces lactate production, increases ATP production, reduction in glucose consumption and increase in the rate of oxidative phosphorylation relative to glycolysis (OCR/ECAR ratio) in colorectal cancer cells. Formation of small and poorly perfused tumors.	Pate et al. (2014)
Blocking of Wnt/β-catenin signaling by PGC1α-PPARγ	PDK1	Increases basal and maximal OCR and decreases ECAR; increases cellular ATP levels, glucose uptake, and decreases extracellular lactate levels in hepatocellular carcinoma cells. Reversion of the migratory and invasive activity (Zuo et al., 2021).	Zuo et al. (2021)
Blocking of Wnt/β-catenin signaling by Chibby	PDK1	Reduces cellular glucose uptake and lactate production, decreases cellular ATP levels, and increases cellular O_2_ consumption rate in nasopharyngeal cancer cells. Reduces cell proliferation both *in vitro* and *in vivo*.	Cai et al. (2018)
Activation of Wnt/β-catenin/c-Myc by EHD1/14-3-3ζ	HK2, PFKL, PGK1, ENO1, LDHA and PDK1	Higher glucose uptake, ECAR and SUVmax. EHD1/14-3-3ζ/β-catenin/c-Myc positive feedback circuit potentiates non-small cell lung cancer cells proliferation *in vitro* and *in vivo*.	Huang et al. (2021)
Blocking of Wnt/β-catenin signaling by SLC25A18	PKM2, LDHA	Reduces glucose uptake, lactate production and ATP generation. Upregulation of SLC25A18 inhibits cell proliferation and its downregulation promotes cell growth.	Liang et al. (2020)
Blocking of Wnt/β-catenin pathway by SMAR1	GLUT1, PFK	Reduces levels in G6P, glucose consumption, lactate, ATP production, OCR, ECAR and glycolytic flux. Reduction of the viability, proliferation, migration, invasion and EMT of bladder cancer cells.	Bao et al. (2023)
Activation of Wnt/β-catenin pathway by PDLIM1	HK2	Promotion of Warburg effect. PDLIM1 inhibition reduces gastric cancer cell proliferation, migration and invasion, and promotes cell apoptosis.	Lei et al. (2024)
Activation of Wnt/β-catenin/c-Myc pathway	PDK1, MCT-1	MCT-1/SLC16A1 is regulated by Wnt/β-catenin signaling in colon cancer cells. MCT-1 can export lactate, pyruvate as well as a glycolysis-targeting cancer drug, 3-bromopyruvate (3-BP).	Sprowl-Tanio et al. (2016)
Blocking of Wnt/β-catenin pathway by PRI-724 and IWP-O1	PFKM, PKM2 and LDHA	Decreases the expression of glycolytic enzymes and attenuates the survival of tongue carcinoma cells, reduces glucose absorption and lactate release.	Kleszcz et al. (2023)
Blocking of Wnt/β-catenin pathway by KYA1797K or APC^min/+^	PKM2, LDHA	APC-loss causes the increased expression of metabolic genes including PKM2, LDHA and increases glucose consumption and lactate secretion. Warburg effect and growth of xenografted tumors-induced by APC-mutated-colorectal cancer cells were suppressed by PKM2-depletion.	Cha et al. (2021)
Activation of Wnt/β-catenin by Rspo2-LGR4	GCK, PKM, PFKL, G6PD and LDHA	Increases glucose consumption and lactate production. LGR4 could promote hepatocellular carcinoma formation in mouse model induced by DEN and CCl4.	Bi et al. (2024)
Activation of non-canonic Wnt3a-LRP5-RAC1-mTORC2	GLUT1, HK2, PFK1, PFKFB3 and LDHA	Increases the concentration of lactate, glucose consumption and ECAR but not OCR. Reprogramming of glucose metabolism specifically contributes to Wnt-induced osteoblast differentiation.	Esen et al. (2013)
Activation of non-canonic Wnt3a-PI3K/AKT-PFKP	PFKP	Promotion of the Warburg effect, cell proliferation, colony formation and the migratory ability of cancer cells.	Jeon et al. (2021)

This table outlines how specific interventions in Wnt signaling, including inhibitors and activators, modulate the expression of key metabolic enzymes and transporters involved in glycolysis, oxidative phosphorylation, and lactate metabolism. These changes result in altered energy production, glucose uptake, lactate secretion, and metabolic reprogramming, with direct implications for tumor growth, cell proliferation, migration, invasion, and apoptosis. The data presented are supported by experimental evidence from studies conducted across various cancer cell types and models, as referenced.

Beyond conventional nutrient uptake mechanisms, Wnt signaling has been shown to regulate macropinocytosis. This nutrient-scavenging process enables tumor cells to engulf extracellular proteins and break them down into amino acids via lysosomal degradation (Rainero, 2024; Commisso et al., 2013). While glycolysis and glutaminolysis serve as primary metabolic pathways, macropinocytosis acts as an alternative nutrient source under metabolic stress, particularly in hypoxic or glucose-deprived tumor microenvironments (Lambies et al., 2024). Wnt-driven activation of Rac1 and Mechanistic Target of Rapamycin Complex 2 (mTORC2) enhances actin cytoskeleton remodeling, promoting macropinocytic uptake (Tsujimura et al., 2016; Esen et al., 2013). This adaptation allows cancer cells to maintain biosynthesis and proliferation even when key metabolic pathways are restricted (Xu et al., 2025). Although macropinocytosis is not a primary metabolic pathway, its activation in Wnt-driven cancers enhances tumor survival under nutrient-poor conditions (Choi et al., 2017). This has led to studies in therapeutically targeting macropinocytosis alongside glycolysis and glutaminolysis to disrupt tumor metabolic flexibility and inhibit cancer progression (Li et al., 2025; Tejeda-Muñoz et al., 2024). Another emerging link between Wnt signaling and immune evasion is the Wnt5a-driven upregulation of indoleamine 2,3-dioxygenase 1 (IDO1) (Sun G. et al., 2021; Holtzhausen et al., 2015; Zhao et al., 2018). IDO1 is an immunosuppressive enzyme that catabolizes tryptophan into kynurenine, leading to T-cell suppression and immune escape (Zhai et al., 2020). Notably, kynurenine-mediated tryptophan depletion also triggers metabolic shifts, reinforcing glutaminolysis and macropinocytosis as compensatory pathways in Wnt-driven tumors. This metabolic-immune interplay enhances tumor progression and resistance to immunotherapy. Targeting IDO1 in combination with metabolic inhibitors offers a promising therapeutic avenue, as IDO1 blockade (using Epacadostat, Navoximod, Indoximod, etc.) may restore anti-tumor immunity while disrupting metabolic adaptation. This highlights the need for integrated therapeutic strategies which simultaneously targets metabolism and immune evasion in Wnt-driven cancers. Furthermore, the Wnt pathway has been shown to be modulated by key metabolic enzymes and intermediates, sometimes as part of a positive feedback loops (Liang et al., 2023; Nath et al., 2015; Lai et al., 2017; Li et al., 2019; Coant et al., 2010; Madsen et al., 2024; Li et al., 2021; Park et al., 2016; Lu et al., 2021; Wang Z. et al., 2024). Its activation or inhibition depends on the metabolic state of the cell, and this regulation influences both normal physiological processes and altered pathological states (Table 2).

**TABLE 2:** Effects of metabolic intermediates and enzymes on Wnt/β-catenin signaling**.**

Enzymes and metabolic intermediates	Effect on Wnt	Mechanism	References
Butyrate	Inhibits Wnt	Butyrate reduced nuclear β-catenin	Liang et al. (2023)
FAA (High)	Activates Wnt	Stabilizes β-catenin. Increases Wnt gene expression.	Nath et al. (2015)
MUFAs (High)	Activates Wnt	MUFA, generated by Wnt-dependent SCD, provides a positive feedback loop to stabilize β-catenin	Lai et al. (2017)
ROS (High)	Inhibits Wnt	Targeting GLS1 triggers an increase of ROS, attenuates nuclear translocation of β-catenin	Li et al. (2019)
ROS (High)	Inhibits Wnt	Oxidative stress causes dissociation of NRX from Dvl, which enables Dvl to activate the downstream Wnt signalling pathway.	Coant et al. (2010)
AMPK	Activates Wnt	Inhibition of AMPK stabilizes β-catenin.	Madsen et al. (2024)
AMPK	Inhibits Wnt	Increased AMPK activity reduces GSK3β activity.	Li et al. (2021)
AMPK	Inhibits Wnt	Increased AMPK activity reduces nuclear localization of β-catenin.	Park et al. (2016)
PKM2 (Piruvate Kinase M2)	Activates Wnt	β-catenin and the downstream target genes/proteins c-Myc and Cyclin-D1 were upregulated after overexpression of PKM2	Lu et al. (2021)
PC (Piruvate Carboxylase)	Activates Wnt	Overexpression of PC stimulates Wnt/β-catenin pathway. The translocation of β-catenin into the nucleus was prevented by PC knockdown	Wang et al. (2024a)

The Wnt pathway has been shown to be regulated by various branches of cellular metabolism, including intermediates from lipid, mitochondrial, glycolytic, and anaplerotic metabolism. These modulations sometimes form part of a positive feedback mechanism, in which the intermediate or enzyme activates Wnt signaling to increase its concentration or activity, respectively. These changes result in alterations in energy production, glucose uptake, and maintenance of the stem-like phenotype, with direct implications for fibrosis, tumor growth, cell proliferation, migration, invasion, and epithelial-mesenchymal transition.

Given the metabolic plasticity of Wnt-driven tumors, single-agent metabolic inhibitors often fail to induce durable responses. To address this challenge, nanomedicine offers an opportunity to co-deliver multiple metabolic inhibitors with enhanced selectivity and bioavailability (Liu P. et al., 2022). Liposomes, lipid nanoparticles (LNPs), polymeric nanoparticles, and exosome-based drug carriers have shown potential in tumor-targeted delivery of glycolysis inhibitors (Lactate Dehydrogenase A or LDHA, PDK1) (Wang et al., 2020), glutaminolysis inhibitors (Glutaminase or GLS) (Wang Q. et al., 2024), macropinocytosis inhibitors, and IDO1 blockade agents (Jiang et al., 2024). By exploiting tumor-specific metabolic markers (e.g., Glucose Transporter protein type 1 or GLUT1, macropinocytic vesicles), nanocarriers can improve drug accumulation in the tumor microenvironment while minimizing systemic toxicity (Lo et al., 2022). In this review, we discuss the interplay among Wnt signaling, metabolic reprogramming, and immune evasion in colorectal cancer. These insights open new avenues for multi-pronged therapeutic strategies targeting these converging pathways.

The Wnt signaling pathway is highly conserved across all metazoans and regulates critical processes during embryonic development as well as in adult tissue homeostasis (Holstein et al., 2011; Loh et al., 2016; Adamska et al., 2010; Hobmayer et al., 2000; Holstein, 2012). The Wnt1 gene, originally named Int-1, was first identified in 1982 by Nusse and Varmus as a proto-oncogene (Nusse and Varmus, 1982). Since then, at least 19 Wnt family proteins have been identified in mammals. These proteins are essential for early developmental events, including primary body axis formation (Shi, 2024), morphogenetic movements, and germ layer specification during gastrulation (Bubna-Litic et al., 2024). The importance of Wnt signaling in embryogenesis is underscored by the fact that its disruption leads to severe developmental defects or early embryonic lethality (McMahon and Moon, 1989; Zeng et al., 1997; Zylkiewicz et al., 2014; Haegel et al., 1995; Huelsken et al., 2000; Liu et al., 1999). Beyond development, Wnt signaling is involved in the maintenance and regeneration of multiple adult tissues, including cardiac (Li et al., 2022), hepatic (Zou and Park, 2023), follicular (Fu et al., 2024), pulmonary (Raslan and Yoon, 2020), bone (Leucht et al., 2019), dental (Kornsuthisopon et al., 2022), and intestinal tissues (Cordero and Sansom, 2012). It is also critical for maintaining both stem cell identity and tumorigenic phenotypes (Nusse and Varmus, 2012; Mohammed et al., 2016; Yang et al., 2016). Moreover, Wnt signaling regulates genomic stability (Augustin et al., 2017; Malki et al., 2020; Arnovitz et al., 2022), cell fate and differentiation (Bagchi and MacDougald, 2021; Wang J. et al., 2024), proliferation (Lou et al., 2023; Liu et al., 2023; Gaowa et al., 2024), motility (Guo et al., 2021; VanderVorst et al., 2023; Zhang et al., 2022), as well as cell death pathways such as apoptosis and autophagy (Ma et al., 2023).

The Wnt pathway is initiated by the binding of secreted Wnt ligands—cysteine-rich, glycosylated, and acylated proteins of ∼40 kDa (Tanaka et al., 2002; Liu Y. et al., 2022; Barrott et al., 2011; Biechele et al., 2011; Coombs et al., 2010)—to receptor complexes on the target cell surface. These complexes typically consist of a seven-pass transmembrane Frizzled (FZD) receptor and a co-receptor, Low Density Lipoprotein Receptor-Related Protein (LRP) 5 or 6 (Carmon and Loose, 2010; Dijksterh et al., 2015; Hsieh et al., 1999; Voloshanenko et al., 2017). Additionally, Wnt ligands may bind to alternative receptors such as the Receptor Tyrosine Kinase-like Orphan Receptors ROR1 and ROR2 (Mas et al., 1992; Masiakowski and Yancopoulos, 1998; Xu and Nusse, 1998; Yu et al., 2016). In humans, at least 10 distinct FZD receptors have been characterized (Chae and Bothwell, 2018), all featuring an extracellular N-terminal domain with a conserved cysteine-rich domain (CRD), seven transmembrane domains, and an intracellular C-terminal domain (Zheng and Sheng, 2024; Liu et al., 2024). The CRD (approximately 120 amino acids) serves as a key ligand-binding region for Wnt proteins (Janda et al., 2012).

Ligand-receptor specificity largely depends on the particular FZD involved (Hsieh et al., 1999; Voloshanenko et al., 2017). However, mapping definitive Wnt–FZD interactions remains a challenge due to the presence of 19 Wnt and 10 FZD paralogs in mammals and the observation that individual Wnt ligands can bind multiple FZDs, and *vice versa*. The LRP5/6 co-receptor facilitates ligand-receptor complex stabilization and appears to contribute to ligand specificity (He et al., 2004). ROR1 and ROR2 are single-pass transmembrane proteins that contain an extracellular immunoglobulin-like domain, a CRD, and a Kringle domain (KRD); in which the CRD is the principal domain mediating Wnt ligand interaction (Mas et al., 1992; Menck et al., 2021; Saldanha et al., 1998).

**FIGURE 1:**
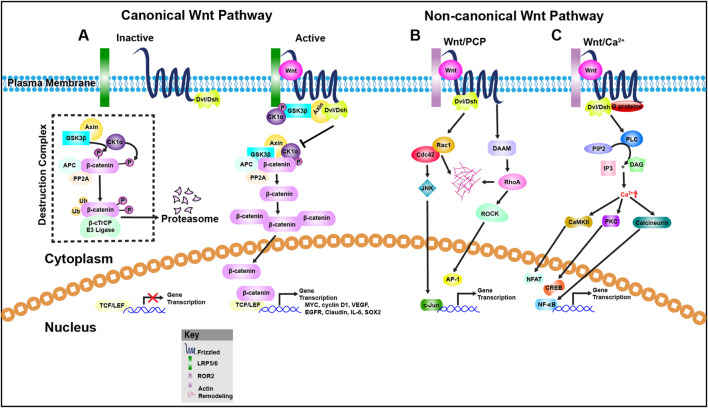
Wnt signaling pathways. **(A)** Canonical Wnt signaling pathway: In the absence of Wnt ligands, β-catenin is targeted for degradation by a destruction complex composed of Axin, APC, CK1α, and GSK3β. This complex phosphorylates β-catenin, marking it for ubiquitination by the β-TrCP E3 ligase and subsequent proteasomal degradation. As a result, β-catenin levels remain low in the cytoplasm, and TCF/LEF transcription factors are bound to corepressors, preventing Wnt target gene expression. Upon Wnt ligand binding to Frizzled (Fz) receptors and co-receptors LRP5/6, Dishevelled (DVL) is recruited to the membrane complex, leading to inhibition of GSK3β and stabilization of β-catenin. Accumulated β-catenin translocates into the nucleus, where it displaces co-repressors and interacts with TCF/LEF to drive transcription of Wnt-responsive genes such as MYC, Cyclin D1, VEGF, EGFR, Claudin, IL-6, and SOX2. **(B)** Non-canonical Wnt/Planar Cell Polarity (PCP) Pathway: This β-catenin–independent pathway regulates cytoskeletal organization and cell polarity. Wnt ligands such as Wnt5a engage Fz receptors and ROR1/2 or RYK co-receptors, activating DVL and downstream small GTPases like Cdc42, Rac1, and RhoA. Rac1 activates JNK signaling, promoting actin remodeling and transcription via AP-1 and c-Jun. RhoA activates ROCK (Rho-associated kinase), contributing to cytoskeletal dynamics and potentially transcriptional regulation. **(C)** Non-canonical Wnt/Calcium (Ca^2+^) Pathway: This pathway is triggered by Wnt ligands such as Wnt5a, Wnt11, or Wnt4, which bind Fz or ROR receptors and activate heterotrimeric G proteins. These in turn stimulate phospholipase C (PLC), leading to hydrolysis of PIP2 into IP_3_ and DAG. IP_3_ induces Ca^2+^ release from the endoplasmic reticulum, which activates CaMKII and calcineurin. Calcineurin dephosphorylates NFAT, promoting its nuclear import and transcriptional activity. DAG activates PKC, which influences other transcription factors including CREB and NF-κB, contributing to diverse cellular responses such as motility, differentiation, and inflammation.

Wnt signaling is broadly categorized into two branches: the β-catenin-dependent (canonical) pathway and β-catenin-independent (non-canonical) pathway (Figure 1). The non-canonical branch includes the Planar Cell Polarity (PCP) pathway and the Wnt/Ca^2+^ pathway (Hayat et al., 2022; Qin et al., 2023). The canonical Wnt/β-catenin pathway is defined by the stabilization and nuclear translocation of β-catenin following Wnt ligand binding (Moon et al., 2004; Rim et al., 2022). In contrast, the PCP pathway governs cellular and tissue polarity along the body axis (Shi, 2022), while the Wnt/Ca^2+^ pathway involves intracellular calcium flux and activation of calcium-sensitive signaling molecules (De, 2011).

Although these pathways were initially considered distinct, recent evidence suggests that Wnt signals may activate multiple pathways concurrently and even antagonistically (Thrasivoulou et al., 2013; Tufail and Wu, 2023). Traditionally, Wnt1, Wnt3a, Wnt8, and Wnt8b have been classified as canonical ligands, whereas Wnt4, Wnt5a, and Wnt11 are linked to non-canonical signaling. However, this classification is now viewed as oversimplified, as growing evidence shows that the same Wnt ligand can activate both canonical and non-canonical pathways depending on the context (Flores-Hernández et al., 2020). We explore the interplay among Wnt signaling, metabolic reprogramming, and immune evasion in colorectal cancer, focusing on key metabolic pathways and delivery strategies that may enhance therapeutic efficacy.

## Canonical Wnt/β-catenin pathway

In the absence of Wnt ligands, the pathway remains inactive due to the continuous activity of the β-catenin destruction complex, which keeps cytoplasmic and nuclear β-catenin levels low (Figure 1A). This multiprotein complex includes the scaffold protein Axin, the tumor suppressor Adenomatous Polyposis Coli (APC), and the serine/threonine kinases CK1α (Casein Kinase 1 alpha) and Glycogen Synthase Kinase 3 beta (GSK3β). β-catenin is sequentially phosphorylated at specific residues (Ser33, Ser37, and Thr41), which marks it for recognition by the E3 ubiquitin ligase complex component β-TrCP. Ubiquitinated β-catenin is then targeted for proteasomal degradation (Hayat et al., 2022). Upon Wnt ligand binding to the FZD-LRP5/6 receptor complex, the intracellular protein Dishevelled (Dvl) is recruited to the membrane and activated. Concurrently, the LRP5/6 co-receptor is phosphorylated. These events lead to the assembly of a receptor-associated “signalosome” that sequesters components of the β-catenin destruction complex, thereby inhibiting GSK3β activity. As a result, β-catenin escapes degradation, accumulates in the cytoplasm, and translocated into the nucleus. In the nucleus, β-catenin binds to T-cell factor/lymphoid enhancer-binding factor (TCF/LEF) family transcription factors to activate Wnt target gene expression involved in proliferation, differentiation, and stem cell maintenance (Hayat et al., 2022).

## Non-canonical Wnt/planar cell polarity (PCP) pathway

The PCP pathway represents a β-catenin-independent branch of Wnt signaling that is highly conserved and essential for directing cellular orientation and movement during morphogenesis (Goodrich and Strutt, 2011; Koca et al., 2022). “Planar polarity” refers to the coordinated alignment of cells within the plane of a tissue orthogonal to the apical-basal axis. The PCP pathway is critical in numerous developmental processes such as gastrulation, neural tube closure, and organogenesis (Williams and Solnica-Krezel, 2020; Henders et al., 2018; Matsuda and Sokol, 2021; Shi, 2023). Mutations in PCP genes are associated with developmental disorders including spina bifida (Bosoi et al., 2011), hearing loss (Sun H. et al., 2021), cystic kidney disease (Richards et al., 2019), and limb malformations (Zhu et al., 2023).

PCP signaling modulates cytoskeletal dynamics through downstream effectors including RhoA, Rac1, JNK, and PKC (Figure 1B), influencing cellular morphology and motility (Wallingford, 2012; Adler and Wallingford, 2017). The pathway relies on a group of evolutionarily conserved core PCP proteins: Frizzled (FZD), Dishevelled (Dvl), Celsr, Vangl, Prickle, and Ankrd6 (Strutt and Strutt, 2009). These proteins form asymmetric complexes on opposite sides of a cell, establishing polarity before any visible morphological changes occur. Wnt ligands such as Wnt5a, Wnt5b, and Wnt11 activate PCP signaling by binding to FZD receptors in complex with ROR1/2 or RYK co-receptors (Gao, 2012). Downstream effectors can be broadly classified into two groups: (1) the planar polarity effectors (PPE), including Daam1, small Rho GTPases, and JNK; and (2) the ciliogenesis and planar polarity effectors (CPLANE), such as Intu, Fuz, and Wdpcp, which are less well characterized but contribute to ciliary assembly and polarity (Goodrich and Strutt, 2011; Yang and Mlodzik, 2015).

## Non-canonical Wnt/calcium pathway

The Wnt/Ca^2+^ pathway is another β-catenin-independent signaling route that regulates intracellular calcium flux and associated cellular functions (Slusarski et al., 1997; Anastas and Moon, 2013). Upon ligand binding—typically by Wnt5a or Wnt11—G proteins are activated, leading to the stimulation of phospholipase C (PLC). This, in turn, generates inositol trisphosphate (IP_3_), which induces the release of calcium from intracellular stores. The resulting calcium surge activates several calcium-dependent effectors, including protein kinase C (PKC) and Ca^2+^/calmodulin-dependent kinase II (CaMKII) (Kühl et al., 2000; Wang et al., 2010), thereby influencing cell migration, polarity, and fate determination (Figure 1C).

A key transcriptional component of the Wnt/Ca^2+^ pathway is the Nuclear Factor of Activated T-cells (NFAT), which is activated via the calcium-dependent phosphatase calcineurin. Upon dephosphorylation, NFAT is translocated to the nucleus where it drives the expression of genes involved in morphogenesis (Farrera-Hernández et al., 2021), mesenchymal-to-epithelial transitions (Dejmek et al., 2006), and immune regulation (Crabtree and Olson, 2002).

## Wnt/β-catenin signaling and aerobic glycolysis

**FIGURE 2:**
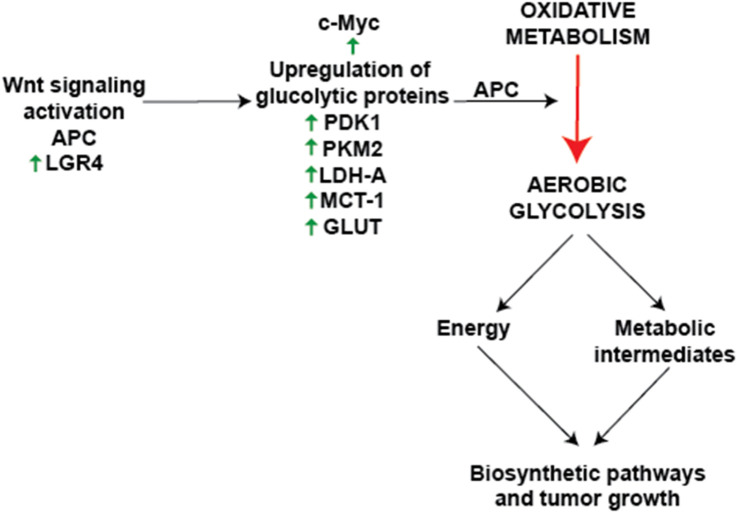
Canonical Wnt signaling and cancer metabolic reprogramming activation of Wnt signaling via the β-catenin pathway leads to the transcriptional regulation of key metabolic enzymes and transporters, including PDK1, LDHA, PKM2, and GLUT1. These molecular changes promote the Warburg effect (aerobic glycolysis), characterized by increased glucose uptake, lactate production, and diversion of metabolic intermediates into biosynthetic pathways critical for tumor growth. The schematic also highlights the inhibition of pyruvate oxidation through the PDK1-mediated inactivation of PDH and its impact on oxidative phosphorylation. Additionally, the role of downstream targets such as c-Myc and LGR4 in supporting glycolytic flux and cell proliferation is depicted. These processes collectively underscore the significance of Wnt signaling in driving metabolic adaptability and cancer progression. This representation summarizes findings on the interrelation between Wnt signaling and cancer metabolism, emphasizing therapeutic opportunities in targeting these pathways.

**FIGURE 3:**
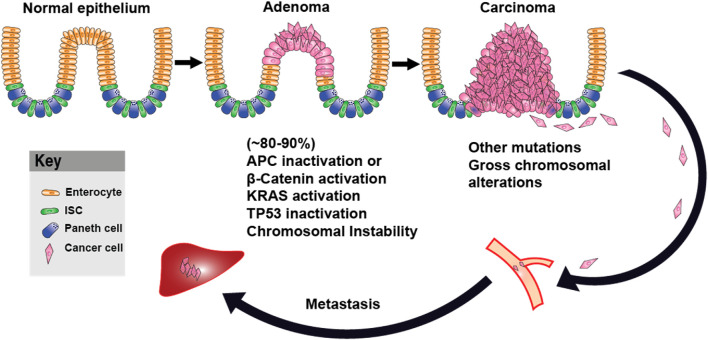
Adenoma–Carcinoma–Metastasis Progression in Colorectal Cancer. Schematic representation of colorectal cancer progression, beginning with normal intestinal epithelium. Loss of APC or activation of β-catenin leads to adenoma formation, followed by the accumulation of additional oncogenic mutations, including KRAS and TP53, and the development of chromosomal instability. These events promote the transition to carcinoma. Further genomic alterations support metastasis. ISC: Intestinal Stem Cell.

Numerous studies support a relationship between Wnt/β-catenin signaling and metabolic reprogramming, particularly aerobic glycolysis. Knockdown assays in lung adenocarcinoma cells have shown that Wnt/β-catenin activation increases c-Myc and PDK1 protein levels, reinforcing its role in enhancing glycolytic flux (Huang et al., 2021). In colon cancer cell lines, overexpression of the mitochondrial glutamate transporter SLC25A18 reduced glucose uptake and downregulated β-catenin, Lactate Dehydrogenase A (LDHA), and Pyruvate Kinase M2 (PKM2), while its deletion had the opposite effect. Notably, the inhibitory effects of SLC25A18 on glucose metabolism, lactate production, ATP generation, and cell proliferation were reversed by the Wnt inhibitor Dickkopf-related protein 1 (DKK1) (Liang et al., 2020). Similar observations were seen in bladder cancer, where overexpression of SMAR1 (Scaffold/matrix attachment region binding protein 1) reduced glycolysis by inhibiting GLUT1 and phosphofructokinase (PFK) expression. Treatment with XAV-939 (a Wnt/β-catenin inhibitor) reversed the increase in glycolytic activity following SMAR1 silencing, while treatment with LiCl (a Wnt activator) negated the effects of SMAR1 overexpression (Bao et al., 2023). In gastric cancer cells, PDLIM1 (PDZ and LIM domain protein 1) interacted with HK2 (Hexokinase 2) to drive glycolysis, proliferation, migration, and apoptosis resistance via Wnt/β-catenin signaling, particularly under glucose-deprived conditions (Lei et al., 2024). Additionally, monocarboxylate transporter 1 (MCT-1), encoded by SLC16A1, was identified as a direct Wnt target gene, and its Wnt response elements (WREs) in the SLC16A1 promoter were sensitive to Wnt inhibition (Sprowl-Tanio et al., 2016). Further supporting Wnt’s role in metabolic control, Wnt inhibitors PRI-724 and IWP-O1 attenuated glucose uptake and lactate production in tongue carcinoma cells while also reducing the expression of Phosphofructokinase, Muscle type (PFKM), PKM2, and LDHA (Kleszcz et al., 2023). Likewise, in colon adenocarcinoma cells, loss of APC increased the expression of PKM2 and LDHA, along with glucose consumption and lactate secretion. Depletion of PKM2 suppressed glycolysis and inhibited xenograft tumor growth in APC-mutant cells (Cha et al., 2021). Beyond APC-driven metabolic shifts, leucine-rich repeat-containing G protein-coupled receptor 4 (LGR4), a receptor in the Wnt pathway, promoted metabolic reprogramming in hepatocellular carcinoma (HCC) by enhancing glycolysis and lactate production. Notably, LGR4 activation by R-spondin ligands enhanced Wnt/β-catenin signaling, driving cancer progression (Bi et al., 2024; Glinka et al., 2011). LGR5, along with its homologs LGR4 and LGR6, belongs to subgroup B of the leucine-rich repeat-containing G protein-coupled receptors (LGRs), a subset of the GPCR superfamily typically associated with hormone signaling (Hsu et al., 2000). While LGR4/5/6 share a high degree of structural similarity, their expression patterns only partially overlap, and functional studies indicate that they have distinct phenotypic effects (Kriz and Korinek, 2018). Although all three receptors are known to modulate Wnt signaling in cooperation with R-spondins, only LGR4 has been conclusively implicated in metabolic regulation via Wnt pathway interactions so far (Zhang et al., 2023). Additionally, c-Myc and cyclin D1, two downstream Wnt targets, further stimulate aerobic glycolysis (Wise et al., 2008). The integration of Wnt signaling with metabolic reprogramming is crucial for cancer progression, particularly through the regulation of glycolytic enzymes such as 3-phosphoinositide-dependent protein kinase 1 (PDK1), PKM2, and LDHA (Rong et al., 2024; Leung and Lee, 2022). The Warburg effect, driven by Wnt signaling, not only enhances energy production but also generates biosynthetic intermediates essential for tumor growth and survival (Figure 2). This metabolic reprogramming is closely associated with the stepwise progression from normal intestinal epithelium to carcinoma and metastasis, as shown in Figure 3. Loss of APC or activation of β-catenin initiates adenoma formation, followed by mutations in KRAS and TP53, and an increase in chromosomal instability, which collectively drive the transition from adenoma to carcinoma. Additional mutations and chromosomal alterations facilitate the metastasis process. Therapeutically targeting Wnt-driven metabolic enzymes thus offers a compelling strategy for malignancies dependent on metabolic reprogramming.

## Non-canonical Wnt pathway and metabolic regulation

**FIGURE 4:**
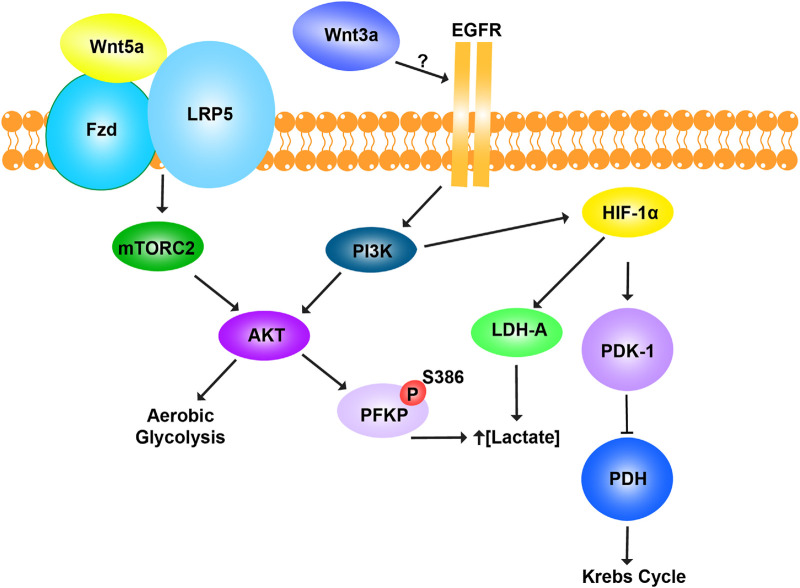
Non-canonical Wnt pathway and metabolic regulation. The Wnt pathway regulates glycolysis and mitochondrial respiration in a β-catenin-independent manner. Wnt5a promotes aerobic glycolysis through activation of the mTORC2-AKT pathway, the co-receptor LRP5. Activation of AKT enhances glycolytic metabolism to meet cellular energy demands. Wnt3a regulates cellular metabolism through a distinct β-catenin-independent mechanism by engaging EGFR, though the exact interaction remains unclear. Wnt3a promotes lactate production by phosphorylating phosphofructokinase platelet type (PFKP) at residue S386 and activating lactate dehydrogenase A (LDH-A). This increases lactate concentration, supporting glycolytic flux. Simultaneously, Wnt3a inhibits mitochondrial respiration by upregulating pyruvate dehydrogenase kinase-1 (PDK-1), which phosphorylates and inactivates pyruvate dehydrogenase (PDH). As a result, pyruvate is diverted away from the Krebs cycle and toward fermentation, enhancing glycolysis. Together, Wnt5a and Wnt3a reprogram cellular metabolism to favor glycolysis, bypassing mitochondrial respiration through β-catenin-independent pathways.

β-catenin-independent (non-canonical) Wnt signaling also plays a key role in energy metabolism regulation (Figure 4). Although Wnt3a is classically characterized as a canonical Wnt ligand that primarily activates the β-catenin–dependent signaling cascade, emerging evidence suggests that, in certain cellular contexts, Wnt3a can also trigger non-canonical signaling responses. Studies have shown that Wnt3a increases aerobic glycolysis by upregulating key glycolytic enzymes. Interestingly, this metabolic regulation requires LRP5 but not β-catenin and instead relies on mTORC2-AKT (also known as Protein Kinase B or PKB) signaling downstream of RAC1 (19). In another assay, Wnt3a-induced EGFR-PI3K/AKT transactivation stabilized phosphofructokinase P (PFKP) via S386 phosphorylation, leading to increased PFKP expression, enhanced glycolysis, as well as cell proliferation and migration, all independent of β-catenin (Jeon et al., 2021). The Wnt pathway also interacts with multiple signaling cascades, including PI3K/Akt, STAT3, and c-Myc-driven transactivation of Hypoxia-Inducible Factor 1-alpha (HIF-1α) (Shih and Mei, 2021). HIF-1α activation promotes aerobic glycolysis, lactate production, and tumor progression, even in normoxic conditions (El-Sahli et al., 2019). Elevated lactate production due to HIF-1α and c-Myc upregulation increases PDK1 and LDHA expression, which enhances glycolytic flux (Liao, 2017). HIF-1α-induced PDK1 expression inhibits the PDH complex, preventing pyruvate entry into the mitochondrial TCA cycle and increasing lactate release. This metabolic shift drives tumor microenvironment remodeling (Gatenby et al., 2006), enhances cell migration (Baumann et al., 2009; Seliger et al., 2013), suppresses the immune response (Fischer et al., 2007; Gottfried et al., 2006), and promotes anti-apoptotic signaling (Kondoh et al., 2005). Additionally, c-Myc and PI3K/Akt-mediated activation of HIF-1α inhibits glucose oxidation via PDK1 and hexokinase 2 (HK2) (Kim et al., 2007; Lum et al., 2007). Thus, non-canonical Wnt signaling not only regulates glycolysis but also modulates key oncogenic pathways such as PI3K/Akt-HIF-1α, shaping the metabolic landscape of cancer cells. These mechanisms underscore new therapeutic targets in metabolic reprogramming, which could complement canonical Wnt inhibition strategies.

## Glutaminolysis and additional metabolic interactions

In cancer cells, pyruvate produced during glycolysis is converted into lactate instead of being utilized in the tricarboxylic acid (TCA) cycle. Although mitochondrial ATP dependency decreases in tumor cells, the demand for biosynthetic precursors and NADPH remains unchanged (Frezza and Gottlieb, 2009). To compensate for these changes and maintain the TCA pathway’s functionality, cancer cells often rely on increased glutaminolysis (Wise and Thompson, 2010; Medina, 2001; Reitzer et al., 1979; Lu et al., 2010). Glutaminolysis is an anaplerotic pathway involving the deamination of glutamine, converting it into glutamate and subsequently into alpha-ketoglutarate (α-KG) by glutaminase (GLS), GDH, and other enzymes. This process enables ATP production through the TCA cycle and provides nitrogen, sulfur, and carbon atoms needed for the synthesis of biosynthetic precursors essential for cancer cell growth and proliferation (Wise and Thompson, 2010; Medina, 2001; Reitzer et al., 1979; Lu et al., 2010). The relationship between Wnt/β-catenin signaling and glutaminolysis remains partially understood; however, emerging evidence highlights key interactions between these two processes. In hepatocellular carcinoma (HCC), overexpression of glutamine synthase (GS) correlates with Wnt/β-catenin activation, indicating a link between glutamine metabolism and tumor progression (Wong et al., 2020). In KRAS-mutated colorectal cancer (CRC) cells, SLC25A22-mediated glutamate transport promotes succinate accumulation, which in turn activates the Wnt pathway, induces DNA methylation, and upregulates LGR5. These effects drive stem cell-like properties, proliferation, and chemotherapy resistance (Cadoret et al., 2002). Additionally, inflammatory responses further integrate glutaminolysis with the Wnt signaling pathway. Pulmonary endothelial cells release R-spondin3, a β-catenin activator, which enhances mitochondrial respiration via glutaminolysis. This produces α-ketoglutarate, which modifies DNA hydroxymethylation via TET2 (Ten-Eleven Translocation-2), linking Wnt activity to epigenetic regulation (Zhou et al., 2020). In gastric cancer (GC) cells, circHECTD1 regulates glutaminolysis via the miR-1256/USP5 axis, activating the β-catenin/c-Myc pathway and promoting tumor progression (Cai et al., 2019). Similarly, niclosamide, a Wnt inhibitor, reduces liver fibrosis and metabolic reprogramming, highlighting potential therapeutic applications (El-Ashmawy et al., 2020). These findings underscore the intertwined relationship between glutaminolysis, epigenetic remodeling, and Wnt signaling in cancer progression and therapy resistance. Targeting Wnt-glutaminolysis interactions may offer novel strategies for disrupting tumor metabolism and enhancing therapeutic efficacy.

## Wnt signaling and macropinocytosis as an adaptive metabolic strategy

**FIGURE 5:**
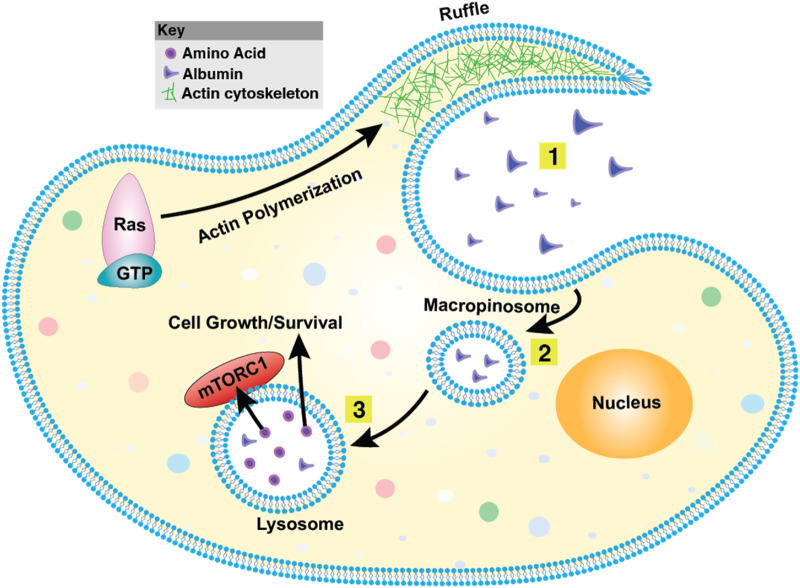
Macropinocytosis overview. (1) The plasma membrane generates actin-driven ruffles, with Ras functioning as a key molecular switch at this early stage. (2) These ruffles fold back onto the membrane, engulfing extracellular fluid and forming large vesicles known as macropinosomes. (3) Macropinosomes undergo maturation and can fuse with endosomes or lysosomes, leading to the degradation of internalized material. This process plays a vital role in nutrient acquisition, antigen sampling, and pathogen entry. The mechanistic Target of Rapamycin Complex 1 (mTORC1) is a central regulator of macropinocytosis, integrating nutrient availability with cell growth and metabolic responses.

Macropinocytosis is a form of endocytosis characterized by the non-selective internalization of extracellular fluid into large vesicles known as macropinosomes (Mercer and Helenius, 2009; Bloomfield and Kay, 2016). These vesicles are typically 0.2–10 μm in diameter—significantly larger than those formed by other endocytic pathways—which enables the uptake of solutes and nutrients that cannot be internalized through more selective mechanisms such as clathrin-mediated endocytosis (Welliver et al., 2011). Once formed, macropinosomes traffic through the endocytic system, where their contents are degraded, and the resulting metabolites are absorbed by the cell (Buckley and King, 2017). The process is driven by actin cytoskeletal remodeling and the formation of membrane ruffles that fold back onto the plasma membrane to engulf extracellular fluid (Welliver et al., 2011; Hoon et al., 2012; Bernitt et al., 2015; Valdivia et al., 2017). Unlike receptor-mediated endocytosis, macropinocytosis is a non-selective process that is typically activated by extracellular signals. It plays critical roles in cellular nutrient acquisition (Amyere et al., 2002), immune surveillance and antigen presentation (Sallusto et al., 1995; Bondy-Denomy et al., 2013; Liu and Roche, 2015; Canton, 2018), microbial pathogenesis (Lee C. H. R. et al., 2019; Lee J. H. et al., 2019), and tumor biology (Figure 5).

First described nearly a century ago (Edwards, 1925; Lewis, 1931), macropinocytosis has since been observed across a wide range of organisms, from amoebae to mammals, suggesting an evolutionarily ancient origin (Guha et al., 2003; Fares and Greenwald, 2001; Ganot et al., 2020). In cancer, macropinocytosis contributes to tumor cell survival, proliferation, and metastasis, particularly under stressful conditions such as hypoxia or nutrient deprivation (Commisso et al., 2013; Kamphorst et al., 2015). Its activation is commonly driven by oncogenic mutations in genes such as *KRAS*, components of the PI3K/AKT pathway, and Rho-family GTPases (Overmeyer et al., 2008; Palm et al., 2015; Kim et al., 2018). Furthermore, aberrant activation of signaling pathways including Wnt (Redelman-Sidi et al., 2018; Albrecht et al., 2020), Hippo (King et al., 2020), and the energy sensor AMPK (Kim et al., 2018) can also induce macropinocytosis in specific cancer contexts. Importantly, this process has been implicated in mechanisms of therapeutic resistance, highlighting its relevance in cancer progression and treatment response (Qian et al., 2014). The convergence of Wnt signaling, macropinocytosis, and metabolic reprogramming is a key adaptive strategy in cancer, allowing tumor cells to thrive under nutrient-limited conditions. Although canonical Wnt/β-catenin signaling is primarily recognized for its role in tumor progression, recent studies reveal its involvement in controlling macropinocytosis, a vital mechanism for sustaining cellular growth. (Tejeda-Muñoz et al., 2019). Macropinocytosis enables tumor cells to engulf extracellular proteins, which are subsequently degraded in lysosomes to provide amino acids and metabolic intermediates, thereby supporting biosynthetic processes, proliferation, and redox homeostasis (Tejeda-Muñoz et al., 2019; Tejeda-Muñoz and Mei, 2024). This pathway is particularly critical in hypoxic and nutrient-deprived tumor regions, where conventional nutrient uptake mechanisms are insufficient to meet the metabolic demands of rapidly proliferating cancer cells (Amyere et al., 2002; Tejeda-Muñoz et al., 2019; Tejeda-Muñoz and Mei, 2024). Recent findings suggest that Wnt signaling enhances macropinocytosis through actin cytoskeleton remodeling, facilitating extracellular protein uptake and metabolic adaptation (Figure 5). By utilizing macropinocytosis, tumor cells maintain metabolic flexibility, allowing them to sustain biosynthesis even under nutrient limitations (Tejeda-Muñoz and Robertis, 2022). Additionally, macropinocytosis buffers oxidative stress by providing amino acids essential for redox homeostasis and modulates the tumor immune response, enabling immune evasion (Shen et al., 2018). Wnt-driven macropinocytosis also interacts with lysosomal function, promoting focal adhesion dynamics that enhance tumor cell motility and invasion (Tejeda-Muñoz and Robertis, 2022). Focal adhesions, which link the actin cytoskeleton to the extracellular matrix (ECM), are dynamically regulated during cancer cell migration and metastasis (Shen et al., 2018). Wnt activation, through both canonical and non-canonical pathways, facilitates ECM remodeling, reinforcing the invasive potential of cancer cells (Penny et al., 2023; Pham et al., 2022).

Macropinocytosis is intricately linked to Wnt-regulated metabolic pathways, including the Warburg effect and glutaminolysis, which enable tumors to maintain high proliferative rates even in hypoxia (Tejeda-Muñoz and Robertis, 2022). The Warburg effect provides ATP through glycolysis, while glutaminolysis supplies TCA cycle intermediates and biosynthetic precursors. Recent studies demonstrate that macropinocytosis and glutaminolysis are co-regulated by Wnt signaling. Specifically, Wnt/β-catenin signaling regulates the expression of glutamine transporters and enzymes, such as glutaminase (GLS), which catalyzes the conversion of glutamine to glutamate (Shen et al., 2021). This highlights the role of Wnt signaling in orchestrating multiple metabolic pathways to support tumor growth.

Beyond canonical Wnt signaling, non-canonical Wnt pathways also contribute to macropinocytosis regulation. Components such as Rac1 and mTORC2 integrate non-canonical Wnt signaling with metabolic adaptation (Tejeda-Muñoz and Mei, 2024). Rac1, a small GTPase, is essential for actin cytoskeleton remodeling and macropinosome formation, facilitating nutrient scavenging in tumor cells (Buckley et al., 2020). mTORC2, a key metabolic regulator, is activated downstream of non-canonical Wnt signaling (Fu et al., 2021) and influences glycolysis, Warburg metabolism, and protein translation (Esen et al., 2013). This crosstalk between Wnt signaling, Rac1, and mTORC2 underscores the metabolic adaptability of cancer cells, where both canonical and non-canonical pathways converge to drive tumor survival.

Targeting Wnt signaling to disrupt macropinocytosis presents a promising therapeutic approach, as it could restrict nutrient availability, impair metabolic flexibility, and inhibit tumor growth. Wnt inhibitors such as PRI-724, which have been investigated for anti-tumor activity, show potential for modulating metabolic pathways and impairing macropinocytosis (Tejeda-Muñoz et al., 2019; Tejeda-Muñoz and Mei, 2024). Cancers with APC mutations or aberrant Wnt signaling may be particularly sensitive to therapies targeting macropinocytosis and associated metabolic rewiring. Additionally, combining Wnt inhibitors with ECM-targeting agents or immune checkpoint inhibitors may enhance therapeutic efficacy by simultaneously disrupting tumor metabolism and the tumor microenvironment. Given that macropinocytosis supports both metabolic adaptation and immune evasion, dual inhibition strategies could provide long-term benefits by overcoming resistance mechanisms.

## Wnt5a-IDO1 axis: linking metabolic reprogramming and immune evasion

Wnt signaling not only regulates metabolic reprogramming but also plays a crucial role in modulating immune evasion (Malladi et al., 2016; Katoh and Katoh, 2022). An emerging link between Wnt signaling and immune suppression is the Wnt5a-driven upregulation of IDO1, an immunoregulatory enzyme that depletes tryptophan and generates immunosuppressive kynurenine metabolites (Holtzhausen et al., 2015). IDO1 activity is known to promote immune tolerance within the tumor microenvironment, enabling evasion of T-cell-mediated surveillance and resistance to immune checkpoint blockade therapy (Zhao et al., 2018). Recent studies have shown that Wnt5a, a non-canonical Wnt ligand, activates the IDO1 pathway, thereby reinforcing immune suppression while sustaining metabolic adaptations that support tumor survival and proliferation (Holtzhausen et al., 2015; Zhao et al., 2018; DeVito et al., 2021). This shift promotes the expansion of regulatory T cells (Tregs) while suppressing the activation of effector T cells (Thaker et al., 2013). In colorectal cancer, aberrant Wnt5a signaling correlates with elevated IDO1 expression and reduced infiltration of cytotoxic CD8^+^ T cells, indicating a more immunosuppressive tumor microenvironment (Thaker et al., 2013). Moreover, tryptophan deprivation or depletion further exacerbates metabolic stress within the tumor microenvironment, reinforcing pathways such as glutaminolysis and macropinocytosis as compensatory mechanisms for nutrient acquisition (Thaker et al., 2013). This metabolic-immune crosstalk highlights how Wnt5a-driven IDO1 activity facilitates immune escape and sustains metabolic plasticity, thereby supporting tumor progression.

The metabolic effects of IDO1 extend beyond immune evasion, influencing key nutrient utilization pathways that sustain tumor cell survival. IDO1-induced tryptophan deprivation/depletion alters amino acid metabolism, shifting cellular dependence toward glutaminolysis as a compensatory energy source (Odunsi et al., 2022). This adaptation is particularly evident in Wnt-driven tumors, where IDO1 activation leads to the upregulation of glutamine transporters (SLC1A5, SLC7A5) and glutaminase (GLS1), enhancing glutamine metabolism to compensate for the lack of tryptophan-derived intermediates (Yoo and Han, 2022). In addition to glutaminolysis, IDO1 activity influences lipid metabolism and mitochondrial function. Studies suggest that kynurenine accumulation promotes oxidative phosphorylation (OXPHOS) and fatty acid oxidation (FAO), favoring metabolism pathways that are less dependent on glucose (Zhao et al., 2018). This metabolic plasticity enables tumors to thrive in nutrient-deprived and hypoxic microenvironments, further reinforcing the survival advantage of Wnt5a-IDO1 signaling. The immunosuppressive effects of Wnt5a-IDO1 signaling pose significant challenges for immunotherapy, particularly in tumors resistant to immune checkpoint inhibitors (ICIs) targeting Programmed Cell Death Protein 1 (PD-1), Programmed Death-Ligand 1 (PD-L1), and Cytotoxic T-Lymphocyte-Associated protein 4 (CTLA-4) (Liu et al., 2021). IDO1-mediated kynurenine accumulation has been shown to suppress dendritic cell maturation and impair antigen presentation, leading to reduced activation of tumor-infiltrating lymphocytes (TILs) (Jochems et al., 2016). Furthermore, Wnt5a signaling promotes the expression of PD-L1 on tumor cells and myeloid-derived suppressor cells (MDSCs), reinforcing an immunosuppressive microenvironment that dampens anti-tumor immunity (Douglass et al., 2021). Clinical studies indicate that tumors with high Wnt5a and IDO1 expression exhibit poor responses to immune checkpoint blockade, suggesting that the Wnt5a-IDO1 axis serves as a mechanism of primary or acquired resistance to immunotherapy. Targeting this pathway alongside ICIs could enhance therapeutic efficacy by restoring anti-tumor immunity and reversing metabolic adaptations that fuel tumor progression (Holtzhausen et al., 2015; DeVito et al., 2021; Theivanthiran et al., 2020). Given the dual role of Wnt5a-IDO1 signaling in immune evasion and metabolic adaptation, therapeutic strategies targeting this axis hold promise for overcoming resistance to immunotherapy and metabolic inhibitors. Pharmacological IDO1 inhibitors such as Epacadostat, Navoximod, and Indoximod have shown potential in reversing kynurenine-mediated immune suppression (Prendergast et al., 2017). However, results from clinical trials, such as ECHO-301, demonstrate that IDO1 inhibition alone or in combination with anti-PD1 is insufficient in highly immunosuppressive tumors (Muller et al., 2019; Chen et al., 2021; Smith et al., 2020). These outcomes underscore the need to explore new combination strategies for IDO1 inhibition. Wnt5a signaling can be targeted using small-molecule Wnt inhibitors (e.g., ETC-159, C59) or monoclonal antibodies blocking Wnt5a-Fzd interactions (Chua et al., 2023; Motono et al., 2016). These inhibitors may not only suppress IDO1 expression but also disrupt Wnt5a-driven metabolic adaptations, thereby sensitizing tumors to immunotherapy and metabolic-modulating therapeutics.

## Advanced therapeutic delivery strategies for immuno-metabolic reprogramming in Wnt-driven cancers

The intricate interplay among Wnt signaling, metabolic plasticity, and immune evasion in tumors presents a significant challenge for conventional therapies. Wnt-driven cancers exploit aerobic glycolysis (the Warburg effect), glutaminolysis, and macropinocytosis to sustain their biosynthetic needs, ensuring tumor survival in nutrient-deprived conditions while evading immune surveillance. Given these complexities, therapeutic delivery strategies must integrate metabolic and immunomodulatory interventions to reprogram the tumor microenvironment and improve treatment efficacy. Advances in nanomedicine, biomaterials, and engineered drug delivery platforms offer new opportunities to co-target metabolic and immune pathways, addressing tumor resistance mechanisms at multiple levels. One promising approach involves co-delivering small-molecule or nucleic acid-based metabolic inhibitors alongside immune-modulating agents using lipid nanoparticles (LNPs) (Wei et al., 2024; Nel et al., 2022), polymeric nanocarriers (Dubey et al., 2017; Mei et al., 2016), and liposomes (Shah et al., 2020; Etter et al., 2021). These nanocarriers can be further functionalized to be immune-stimulatory or immune cell-targeted (Miao et al., 2019; Mei et al., 2023; Mei et al., 2024).

**FIGURE 6:**
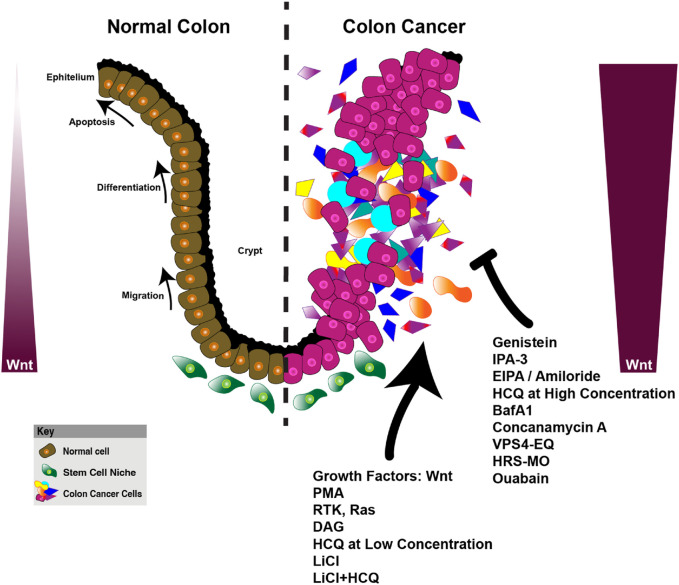
Wnt signaling and its role in colon cancer progression, macropinocytosis, and metabolic reprogramming. This figure compares the organization of the normal colon epithelium and the dysregulated colon cancer microenvironment, highlighting the impact of Wnt signaling on cellular processes. In the normal colon (left), Wnt signaling regulates key biological processes such as apoptosis, differentiation, and migration within the epithelial layer, contributing to homeostasis. The normal stem cell niche is tightly controlled, ensuring proper cellular turnover and balance. On the right, the colon cancer model illustrates the disruption of these processes, with enhanced proliferation, migration, and altered metabolic activity in cancerous cells. Wnt signaling in cancer cells is upregulated, promoting macropinocytosis (phorbol 12-myristate 13-acetate (PMA), RTK, Ras, DAG (diacylglycerol), HCQ at low concentration, LiCl, LiCl+HCQ)) and metabolic reprogramming to support tumor growth. Various compounds targeting Wnt signaling, macropinocytosis (EIPA, Amiloride, Ouabain, Genistein, IPA-3), lysosomes (HCQ at high concentration, Bafilomycin A1, VPS4-EQ (VPS4 ATPase-defective mutant), and hepatocyte growth factor-regulated tyrosine kinase substrate-morpholino oligomers (HRS-MO) are shown as therapeutic interventions that may alter cancer progression. The key shows the differentiation of normal cells (brown), stem cell niche (green), and colon cancer cells (pink and purple), reflecting the complex interplay of signaling pathways and metabolic alterations in cancer progression (Tejeda-Muñoz et al., 2024; Albrecht et al., 2020; Tejeda-Muñoz et al., 2022a; Colozza et al., 2020; Tejeda-Muñoz and De Robertis, 2022; Azbazdar et al., 2023; Tejeda-Muñoz et al., 2022b; Tejeda-Muñoz et al., 2022c; Azbazdar et al., 2024).

Several metabolic enzymes and pathways have emerged as therapeutic vulnerabilities in Wnt-driven tumors, including lactate dehydrogenase A (LDHA), pyruvate dehydrogenase kinase 1 (PDK1), monocarboxylate transporters (MCTs), glutaminase (GLS), and key regulators of macropinocytosis. Given prior evidence that glycolysis inhibition impairs tumor growth and that IDO1 blockade restores anti-tumor immunity, combining these strategies presents a promising but underexplored approach. LNPs delivering siRNA against IDO1 (Endo et al., 2019), or liposomes delivering IDO1 inhibitors (Mei et al., 2020), combined with glycolytic inhibitors such as LDHA or PDK1 inhibitors (Cheng et al., 2024; Lucero-Acuña et al., 2014) may synergistically disrupt tumor metabolic plasticity while reducing immune suppression. Similarly, nanocarriers loaded with glutaminase (GLS) inhibitors, like CB-839 liposomes, could block glutaminolysis, a critical compensatory pathway in highly glycolytic Wnt-driven tumors (Ni et al., 2023; Shen et al., 2024). Since glutamine metabolism supports nucleotide biosynthesis and redox balance, dual nanoparticle-based inhibition of IDO1 and glutaminolysis may prevent tumors from escaping metabolic stress, thereby enhancing anti-tumor efficacy. Beyond targeting glycolysis and glutaminolysis, macropinocytosis inhibition presents an emerging therapeutic strategy in Wnt-driven tumors that rely on nutrient scavenging (Figure 6). Small molecule macropinocytosis inhibitors, such as EIPA (5-(N-Ethyl-N-isopropyl)amiloride) and Rac1/mTORC2 inhibitors, impair the internalization and lysosomal degradation of extracellular proteins, effectively starving tumors that rely on this process (Son et al., 2023). Targeted delivery of macropinocytosis inhibitors in combination with metabolic disruptors could limit both exogenous nutrient uptake and internal metabolic pathways, cutting off key survival mechanisms for tumors. Interestingly, macropinocytosis can also be leveraged as a drug delivery mechanism to enhance therapeutic uptake into tumor cells. Since Wnt-driven tumors exhibit high macropinocytic activity, nanoparticles engineered to mimic extracellular proteins, also known as protein corona (Mei et al., 2018; Li et al., 2024; Mei et al., 2021), or nutrients could facilitate preferential uptake by tumor cells, thereby improving drug bioavailability and selectivity. Albumin-coated nanoparticles could exploit tumor macropinocytosis (Cullis et al., 2017), while PEGylated nanoparticles may evade immune clearance while still being efficiently endocytosed by macropinocytic vesicles (Guo et al., 2023). Charge-modified polymeric nanoparticles can further enhance intracellular retention following micropinocytosis (Means et al., 2022). By hijacking macropinocytosis for selective drug delivery (Jiang et al., 2023; Hyun et al., 2018), nanoparticle formulations could achieve wider tumor accumulation, improve intracellular drug bioavailability, and reduce systemic toxicity. This approach is particularly advantageous for delivering metabolic inhibitors (e.g., LDHA, GLS, or IDO1 blockers) that require efficient cytosolic access to exert their effects.

In addition to nanoparticle-based systems, biomaterial scaffolds and hydrogels offer localized metabolic reprogramming within tumors. Biodegradable hydrogels embedded with IDO1 inhibitors (Zhuang et al., 2023), kynureninase (Wang et al., 2022a), Wnt or GSK-3β antagonists (Wang et al., 2022b), glycolytic inhibitors (LDHA, PDK1) (Meng et al., 2022), and glutaminolysis inhibitors (GLS) (Guo et al., 2025) could provide sustained drug release which gradually alters the tumor microenvironment and preventing metabolic adaptation over time. Stimulus-responsive biomaterials, such as pH- or redox-sensitive hydrogels, can ensure that drug release occurs only in metabolically active, highly glycolytic tumor environments, thereby minimizing off-target effects while maximizing therapeutic efficacy. Another promising approach is exosome-based drug delivery, which offers tumor-specific targeting while minimizing immune clearance. Engineered exosomes can be loaded with siRNA (Amiri et al., 2022), miRNA (Mathiyalagan and Sahoo, 2017), metabolic inhibitors, or Wnt-targeting molecules (e.g., PRR7, 14-3-3ζ) (Lee et al., 2018; Zhang et al., 2016), allowing for precise targeting of Wnt-driven tumors. Unlike synthetic nanoparticles, exosomes have natural biocompatibility and immune-modulating properties, making them attractive candidates for disrupting both metabolic plasticity and immune suppression.

Given the metabolic adaptability of Wnt-driven tumors, single-agent metabolic inhibitors are unlikely to yield durable responses. The integration of nanomedicine, biomaterials, and exosome-based drug delivery presents a promising avenue for multi-targeted tumor reprogramming. Future research should focus on optimizing nanoparticle design, engineering exosomes for metabolic inhibition, and refining biomaterial-based delivery platforms to enhance drug selectivity, reduce systemic toxicity, and improve tumor-specific accumulation. Additionally, identifying metabolic biomarkers that predict treatment response could facilitate precision-targeted therapies that consider both tumor metabolism and immune modulation. With continued advancements in precision medicine, the integration of immuno-metabolic reprogramming with cutting-edge drug delivery may pave the way for more effective, durable, and personalized cancer therapies.

## Conclusion and future perspectives

Wnt-driven cancers exhibit metabolic plasticity that enables their survival in nutrient-deprived conditions while evading immune surveillance. These tumors rely on aerobic glycolysis (the Warburg effect), glutaminolysis, and macropinocytosis to meet their biosynthetic demands and energy requirements. Key metabolic targets include LDHA, PDK1, HK2, GLUT1, and MCT1, which regulate glycolysis and lactate metabolism; GLS, SLC1A5, and SLC7A5, which drive glutaminolysis and glutamine-dependent biosynthesis; as well as Rac1 and mTORC2, which enhance macropinocytosis and extracellular protein scavenging. Additionally, the Wnt5a-IDO1 axis promotes immune evasion by upregulating tryptophan catabolism through IDO1, resulting in kynurenine accumulation and T-cell suppression. Given the compensatory nature of these metabolic pathways, single-agent therapies often fail due to metabolic rewiring. A multi-pronged therapeutic strategy that simultaneously targets glycolysis, glutaminolysis, macropinocytosis, and immune escape mechanisms may be necessary to achieve durable anti-tumor effects. Several metabolic inhibitors have shown promise in preclinical and early-phase clinical studies. Glycolysis inhibitors such as LDHA inhibitors (FX11), PDK1 inhibitors (DCA, AZD7545), and HK2 inhibitors (3-BrPA) have demonstrated efficacy in limiting glucose metabolism and reducing tumor growth. Glutaminolysis inhibitors, including GLS1 inhibitors (CB-839/Telaglenastat, BPTES, DON derivatives), have been investigated for their ability to impair glutamine-dependent tumor metabolism. Macropinocytosis inhibitors such as EIPA (5-(N-Ethyl-N-isopropyl)amiloride), Rac1 inhibitors (EHT 1864), and mTORC2 inhibitors (PP242, Torin2) present novel approaches to starve tumor cells by blocking their nutrient scavenging mechanisms. Given the immunosuppressive role of IDO1, IDO1 inhibitors (Epacadostat, Navoximod, Indoximod) are being explored in combination with immune checkpoint inhibitors (ICIs) such as anti-PD-1 and anti-CTLA-4 therapies to enhance T-cell activation and improve immune responses in Wnt-driven cancers. Advanced therapeutic delivery platforms offer a promising approach to co-delivering metabolic inhibitors and immunotherapies, improving tumor specificity while minimizing systemic toxicity. Lipid nanoparticles (LNPs), polymeric nanocarriers, and exosomes enable the precise delivery of siRNA-based therapies targeting IDO1, PDK1, GLS, or regulators of macropinocytosis. Albumin-coated nanoparticles and PEGylated liposomes can exploit macropinocytosis as a selective uptake mechanism, enhancing drug accumulation in tumors with high macropinocytic activity. Additionally, biomaterial-based hydrogels and tumor-responsive scaffolds offer localized and sustained drug release, thereby enhancing therapeutic efficacy while minimizing off-target effects. Future research could focus on optimizing drug combinations, refining nanocarrier engineering, and identifying metabolic biomarkers to guide the development of precision-targeted therapies. Given the complex interplay between metabolism and immune evasion, combining metabolic inhibitors with immune checkpoint blockade (ICB), IDO1 inhibitors, or Wnt antagonists may yield synergistic therapeutic effects. Stimuli-responsive biomaterials, such as pH- or redox-sensitive hydrogels, could enable tumor-specific drug activation, further enhancing selectivity and minimizing toxicity. Ultimately, integrating metabolic targeting, immune modulation, and advanced drug delivery technologies may pave the way for more effective, durable, and personalized treatment strategies for Wnt-driven cancers. By leveraging these innovations, it may be possible to overcome metabolic adaptability, restore anti-tumor immunity, and improve clinical outcomes in aggressive and therapy-resistant malignancies.
